# Oral nicotine pouches with an aftertaste? Part 2: in vitro toxicity in human gingival fibroblasts

**DOI:** 10.1007/s00204-023-03554-9

**Published:** 2023-07-23

**Authors:** Selina Rinaldi, Elke Pieper, Thomas Schulz, Ralf Zimmermann, Andreas Luch, Peter Laux, Nadja Mallock-Ohnesorg

**Affiliations:** 1 Department of Chemical and Product Safety, German Federal Institute for Risk Assessment (BfR), 10589 Berlin, Germany; 2 Chair of Analytical Chemistry, Joint Mass Spectrometry Centre, University of Rostock, 18059 Rostock, Germany

**Keywords:** Nicotine pouches, Oral in vitro toxicity, Flavor toxicity, Flavor ingredients

## Abstract

Nicotine pouches contain fewer characteristic toxicants than conventional tobacco products. However, the associated risks in terms of toxicity and addiction potential are still unclear. Therefore, endpoints of toxicity and contents of flavoring substances were investigated in this study. The in vitro toxicity of five different nicotine pouches and the reference snus CRP1.1 were studied in human gingival fibroblasts (HGF-1). Cells were exposed to product extracts (nicotine contents: 0.03–1.34 mg/mL) and sampled at different time points. Cytotoxicity, total cellular reactive oxygen species (ROS) levels, and changes in the expression levels of inflammatory and oxidative stress genes were assessed. Flavor compounds used in the nicotine pouches were identified by GC–MS. Cytotoxicity was observed in two nicotine pouches. Gene expression of interleukin 6 (*IL6*) and heme oxygenase 1 (*HMOX1)* was upregulated by one and three pouches, respectively. ROS production was either increased or decreased, by one pouch each. CRP1.1 caused an upregulation of *IL6* and elevated ROS production. Toxicity was not directly dependent on nicotine concentration and osmolarity. A total of 56 flavorings were detected in the five nicotine pouches. Seven flavorings were classified according to the harmonized hazard classification system as laid down in the European Classification, Labelling and Packaging regulation. Nine flavorings are known cytotoxins. Cytotoxicity, inflammation, and oxidative stress responses indicate that adverse effects such as local lesions in the buccal mucosa may occur after chronic product use. In conclusion, flavorings used in nicotine pouches likely contribute to the toxicity of nicotine pouches.

**Supplementary Information:**

The online version contains supplementary material available at 10.1007/s00204-023-03554-9.

## Introduction

Scientifically, the deleterious health effects of tobacco smoke were confirmed already decades ago (Evans 1962). As a consequence of tobacco control strategies, the global tobacco smoking prevalence decreased from 26.9% in 2000 to 17.0% in 2020 (World Health Organization 2021); and it is projected to decline further to 15.4% by 2025 (World Health Organization 2021). In response to this trend, the tobacco industry has developed new nicotine delivery products with and without tobacco leaf material, such as e-cigarettes, heated tobacco products, and nicotine pouches. Nicotine pouches have been introduced to the US and European market in 2016 and 2018, respectively (Delnevo et al. 2021; Tobacco Tactics 2021). The sales increased rapidly by 124% from 2019 to 2020 (Foundation for a Smoke-Free World 2021). This was underlined by a recent survey conducted in the US among current smokers, in which 16.8% of the respondents reported to be interested in trying oral nicotine pouches (Hrywna et al. 2022). A study from the US and another from the Netherlands found out that the most frequent reason for using nicotine pouches was the reduced risk perception in contrast to tobacco products (Havermans et al. 2021; Plurphanswat et al. 2020). Although the age of users pointed toward a more adult population (Hrywna et al. 2022; Plurphanswat et al. 2020), which was likely due to the study design, Havermans and coworkers found that almost 10% of minors were aware of nicotine pouches and 0.3% had tried it. In particular, young adults are being attracted by the availability of a wide range of flavors, which therefore can be judged as a major concern from the health perspective side (Robichaud et al. 2020). Accordingly, nicotine pouches need to undergo toxicological investigations to shed light into possible inherent risks.

The main compositional and active ingredient of nicotine pouches is the nicotine salt itself (Stanfill et al. 2021). Besides nicotine, they contain additives, such as flavorings, sweeteners, humectants, and pH regulators, all wrapped in a pouch made of viscose fibers (Azzopardi et al. 2021; Robichaud et al. 2020). After placement of the nicotine pouch under the lip, the released nicotine is absorbed through the buccal mucosa.

In its appearance and kind of use, nicotine pouches are similar to snus, but in contrast to snus, they are free from tobacco leaf material. Despite containing much less toxicants than cigarette smoke, snus is not to be considered as risk-free (IARC 2007). Carcinogens, such as tobacco-specific nitrosamines (TSNAs), polycyclic aromatic hydrocarbons, and aldehydes, might be also present in this kind of tobacco product (Hoffmann and Djordjevic 1997). Accordingly, oral lesions are frequently associated with snus use and commonly observed at the site of product placement (Binmadi et al. 2022). Although still detectable, the levels of genotoxic TSNAs are much lower in nicotine pouches when compared to snus, as no tobacco leaf material is present in this final product (Mallock et al. 2022).

Although the tobacco industry advertises nicotine pouches as an alternative to conventional tobacco products toward harm reduction (Imperial Brands 2021), their health effects are still unclear. High nicotine contents of up to almost 50 mg/pouch may contribute to the onset of addiction in novice nicotine users or could lead to other negative health effects, for example, on the cardiovascular system (Mallock et al. 2022; Stanfill et al. 2021). Further, the novel products may exert local cytotoxic effects especially in the oral mucosa. Few studies addressed these issues in the past, most of them published by manufacturers on their proprietary products (Aldeek et al. 2021; Bishop et al. 2020; East et al. 2021; Knopp et al. 2022). Only one study was without industry involvement (Shaikh et al. 2022).

Further independent research addressing factors that may affect human health, such as cytotoxicity, and the identification of unknown substances are needed to inform public health professionals and regulators on the risks possibly associated with the consumption of nicotine pouches.

To address potential health risks that are new to nicotine pouches, this study was conducted in two parts. In part 1, 48 nicotine pouches and 2 nicotine-free pouches were assessed for their ingredients and for further substances identified by a GC–MS-based screening approach (Mallock-Ohnesorg et al. 2023). An initial toxicological assessment was performed for the identified substances based on regulatory databases  (Mallock-Ohnesorg et al. 2023). For part 2, which is described in this manuscript, in vitro toxicity in human gingival fibroblasts (HGF-1) was assessed for five different nicotine pouches and the reference snus CRP1.1. The products were extracted with salt-buffered solution, and the cells were exposed for 24 h to the extracts sampled at different time points. Lactate dehydrogenase (LDH) and metabolic activity (MTT) assays were used as a measure of cytotoxicity. The induction of reactive oxygen species (ROS) was measured using the 2’,7’-dichlorofluorescin diacetate assay (DCFDA), and alterations in the gene expression of inflammatory and oxidative stress markers were assessed via quantitative real-time polymerase chain reaction (qRT-PCR). Nicotine concentrations of sample extracts were quantified using a validated LC-DAD method. Flavorings and other substances identified in the tested pouches were discussed with regard to their potential contribution to toxicity. This two-part study was designed to identify potentially problematic constituents of nicotine pouches and to provide preliminary insights into effects of the products on oral cells. The goal was to set a starting point for future in-depth studies on the mechanisms of nicotine pouch toxicity.

## Materials and methods

### Chemicals and reagents

Nicotine of analytical grade (≥ 99%), ammonium acetate (> 99%), ammonia (25%), Hank’s Balanced Salt Solution (HBSS), hydrochloric acid, and sodium hydroxide were obtained from Merck KGaA (Darmstadt, Germany). 2’,7’-Dichlorofluorescin diacetate (DCFDA) was obtained from Thermo Fisher Scientific (Schwerte, Germany). Dulbecco’s Modified Eagle’s Medium (DMEM, P04-03596) and 4-(2-hydroxyethyl)-1-piperazineethanesulfonic acid (HEPES) were obtained from PAN Biotech (Aidenbach, Germany). 3-(4,5-Dimethylthiazol-2-yl)-2,5-diphenyl tetrazolium bromide (MTT reagent) was obtained from Carl Roth GmbH + Co. KG (Karlsruhe, Germany). Milli Q Integral Water Purification System (Merck KGaA, Darmstadt, Germany) was used to prepare ultra-pure water.

### Nicotine pouch samples and reference snus CRP1.1

For the experiments, five nicotine pouches from five different manufacturers and the CORESTA reference snus product CRP1.1 were used. Nicotine pouches were obtained from online retailers. Nicotine contents were determined in a previous study (Mallock et al. 2022) and ranged from 3.8 to 47.4 mg/pouch. They were selected based on nicotine contents and labeled product flavors (see Supplementary Information Table 1) to cover a broad range of nicotine concentrations and flavor categories.

### Sample extraction

As extraction medium and adapted from Delvadia et al. (2012), a solution of HBSS and HEPES was used with pH-modification according to standard artificial saliva (DIN ISO 53160–1 2010). For 1 L of the solution, 9.8 g HBSS were mixed with 975 mL of ultra-pure water and 25 mL of HEPES were added. The pH was adjusted to 6.8 ± 0.2 using 2 M sodium hydroxide or 4 M hydrochloric acid. This extraction medium was chosen over artificial saliva to avoid possible adverse effects on the cells by the enzymes present in saliva (Malpass et al. 2013). The extraction medium was stored at + 4 °C.

Sample extracts were generated for the time points 5, 10, 20, 30, and 60 min to represent different time periods of product use. Per time point, one pouch was immersed into a flask filled with 10 mL extraction medium and was shaken in a Multitron Pro incubation shaker (Infors HT, Bottmingen, Switzerland) at 37 °C and 200 rpm. After the given extraction times, the whole extract was filtered using a syringe filter with a polyethersulfone membrane (0.22 µm, Merck KGaA,), aliquoted and stored at –20 °C.

### Quantification of nicotine concentrations in sample extracts

Nicotine concentrations of extracts were quantified by LC-DAD. Filtered extracts were diluted 1:10 with extraction medium and 1 µL was injected into the LC system (Agilent 1260 Infinity I + II, G7129AR autosampler, G7112BR pump and degasser, G7116AR column oven, G4212B photodiode array detector, all from Agilent Technologies, Santa Clara, CA, USA). Separation was performed at 45 °C on a Gemini column (NX-C18, 3 µm particle size, 150 mm length, 2 mm inner diameter, 110 Å pore size) with a C18 guard column (both Phenomenex, Torrance, CA, USA). Nicotine was identified by comparing the retention time and UV spectra to a standard substance; it was quantified at 260 nm. Flow rate was constant at 0.2 mL/min. Mobile phase A was 5 mM ammonium acetate and ammonia with a pH of 10 and mobile phase B was methanol. The mobile phase gradient started with 5% B for 1.5 min, followed by an increase to 95% B for 0.2 min and a hold until 8.5 min, followed by a decrease to 5% B for 1 min and a final hold for 3.5 min. Total runtime was 12 min. For data acquisition and analysis, the Chromeleon Chromatography Data System (version 7.2.10, Thermo Fisher Scientific, Schwerte, Germany) was used.

Calibration samples were prepared in extraction medium (0.5, 1, 5, 10, 50, 100, 250, 500, 750, and 1000 µg/mL). The method was validated for linearity, accuracy, precision, stability at 4 °C, limit of detection and quantification (see Supplementary Information Table 2).

### Osmolarity of sample extracts

Osmolarity measurements were performed using a semi-micro osmometer type MLA0299 (Knauer, Berlin, Germany). The apparatus was calibrated to 0 mOsm/kg using distilled water and to 400 mOsm/kg using a 400 mOsm/kg sodium hydroxide solution. Sample extracts were diluted 1:1 with cell culture medium without supplements. 150 µL of the diluted samples was examined for osmolarity.

### Cell culturing

Human gingival fibroblasts (HGF-1; ATCC CRL-2014) were cultured in the recommended Dulbecco’s Modified Eagle’s Medium (DMEM), supplemented with 10% fetal bovine serum (FBS), 1% L-glutamine and 1% streptomycin/penicillin. Cells were passaged once a week with 1–1.5 × 10^5^/mL cells per T75 flask. Normal incubation conditions were 37 °C and 5% CO_2_. During culturing and experiments, cell morphology was monitored using light microscopy and morphological changes were recorded using a microscope camera (Axiocam, both from Zeiss, Oberkochen, Germany).

### Metabolic activity and membrane integrity as measures of cytotoxicity

For cytotoxicity testing, lactate dehydrogenase (LDH) (Roche, Basel, Switzerland) and MTT assays were performed. For this, 96-well plates were seeded with 5 × 10^3^ cells/well. Cells were allowed to attach and to grow for 24 h prior to exposure. Sample extracts were diluted 1:1 with DMEM without supplements and phenol red for cell exposure. The cells were exposed to vehicle control, medium control, diluted sample extracts, nicotine control in dissolution medium or the positive control (1% Triton X-100). Following 24 h of exposure, LDH and MTT assays were performed. While the extraction times aimed to mimic product use durations with potentially different extract compositions, the exposure time of 24 h was chosen according to other studies investigating the toxicity of nicotine pouches (Bishop et al. 2020; East et al 2021; Shaikh et al. 2022). This aims at the comparability of study results.

For the LDH assay, the supernatant was removed and transferred into a new *U*-shaped 96-well plate and centrifuged at 125 rpm for 10 min. After centrifugation, 50 µL of the supernatant were transferred into a new flat-bottom 96-well plate. The LDH reaction mixture was freshly prepared and 50 µL of it were added to the supernatant. After 10 min of incubation at room temperature and protected from light, absorbance was measured at 490 nm and 690 nm using an Agilent Biotek Synergy 2 plate reader (Thermo Fisher Scientific, Schwerte, Germany). For the MTT assay, cells were treated with 100 µL 3-(4,5-dimethylthiazol-2-yl)-2,5-diphenyl tetrazolium bromide (MTT reagent) per well and incubated at 37 °C for 1 h. The MTT reagent was removed and 100 µL DMSO were added to each well. After 15 min on a microplate shaker, absorbance was measured at 595 nm and 690 nm with the plate reader.

### Oxidative stress measurement

The 2',7'-dichlorofluorescin diacetate (DCFDA) assay was used to measure cellular oxidative stress. DCFDA becomes highly fluorescent upon oxidation by reactive oxidative species (ROS) to yield 2',7'-dichlorofluorescein (DCF). 1 × 10^4^ cells were seeded into each well of 96-well plates and allowed to settle for 24 h before exposure. Cells were washed with HBSS and treated with 100 µL of 100 µM DCFDA in HBSS per well for 30 min. Cells were washed with HBSS, which was removed after 30 min. Cells were subsequently exposed to vehicle control, medium control, nicotine control in extraction medium, positive control (2 mM hydrogen peroxide), or sample extracts diluted 1:1 with DMEM without supplements and phenol red. After 4 h, the fluorescence was measured at 480 nm excitation and 535 nm emission using an Agilent Biotek Synergy 2 plate reader (Thermo Fisher Scientific, Schwerte, Germany). Four hours of exposure was used to match the time for gene expression of oxidative stress markers.

### Gene expression measurements

For mRNA extraction, 5 × 10^5^ cells were seeded into each well of 6-well plates and allowed to attach for 24 h. Cells were then exposed to diluted 20-min sample extracts for 4 h. The 20 min sample extracts were chosen as it is a common product use duration according to a survey by one product manufacturer (Prasad et al. 2022). A 4 h exposure was used for gene expression experiments as it has been reported that gene expression of IL8 and IL6 have their peak after 4 to 8 h. Subsequently, mRNA was isolated using the NucleoSpin RNA, Mini kit for RNA purification (Macherey Nagel, Düren, Germany). The procedure was performed following the kit’s protocol. The amount and purity of isolated mRNA was determined using NanoDrop 1000 Spectrophotometer (VWR, Radnor, PA, USA).

Reverse transcription was performed using the High-Capacity cDNA Reverse Transcription Kit (Applied Biosystems, Waltham, MA, USA). Amplification of cDNA was performed on a thermocycler (Bio-Rad, Hercules, CA, USA). QRT-PCR was performed on a Quantstudio 3 instrument (Applied Biosystems, Waltham, MA, USA). Beta-actin (*ACTB*) was used as housekeeping gene. The following targets were analyzed in the study upon exposure to sample extracts or vehicle control: Anti-inflammatory and anti-oxidant gene heme oxygenase 1 (*HMOX1*), anti-oxidant gene glutathione peroxidase (*GPx1*), anti-oxidative gene superoxide dismutase 2 (*SOD2*), pro-/anti-inflammatory gene interleukin 6 (*IL6*), pro-inflammatory gene interleukin 8 (*IL8*), pro-inflammatory gene tumor necrosis factor alpha (*TNFα*). For the primer sequences, see Supplementary Information Table 3. The relative gene expression was calculated based on c_T_ values using the ∆∆c_T_ method.

### Flavor screening of nicotine pouches using GC–MS

Screening for unknown substances contained in nicotine pouches was performed in part 1 of this study where the procedure is described in more detail (Mallock-Ohnesorg et al. 2023). In brief, a method using liquid–liquid extraction (LLE) and gas chromatography with mass spectrometric detection (GC/MS) was adapted from Hutzler et al. (2014). Nicotine pouches were submersed in ultra-pure water and extracted with ethyl acetate under acidic conditions (after addition of 0.1 M hydrochloric acid) and basic conditions (after addition of 0.2 M ammonia). A 2 µl aliquot of the organic phase was injected into the GC/MS system and separated on a DB-17 ms capillary column (30 m × 0.25 mm I.D., 0.25 µm film thickness; Agilent Technologies, Waldbronn, Germany). Peaks were identified using the software Mass Hunter Qualitative Analysis version 10.0 (Agilent Technologies, Waldbronn, Germany) and MSD ChemStation version F.01.03.2365 (Agilent, Technologies, Waldbronn, Germany) and three different spectra libraries: NIST spectral library version 11, Flavor & Fragrance Natural & Synthetic Compounds 3 (FFNSC3) library, and an in-house aroma library created with solutions of standard substances. Nicotine was included as a reference to calculate relative retention times (RRTs). For substances that were included in the in-house library, identification was verified using the RRTs (± 0.05).

### Statistics

Statistical analysis was performed using GraphPad Prism 8 (version 8.2.0 for Windows, GraphPad Software, San Diego, CA, USA). Data derived from the MTT, LDH, and DCFDA assays were analyzed using one-way ANOVA comparing exposed groups with vehicle control group. In case of statistical significance, a Dunnett’s multiple comparison test was used as post hoc test. Fold changes derived from qRT-PCR were analyzed using an unpaired, two-tailed t-test comparing exposed groups with vehicle control group. A *p* value of less than 0.05 was considered statistically significant. Three biological replicates with at least three technical replicates were performed for all experiments.

## Results

### Cytotoxicity (MTT, LDH)

**Fig. 1:**
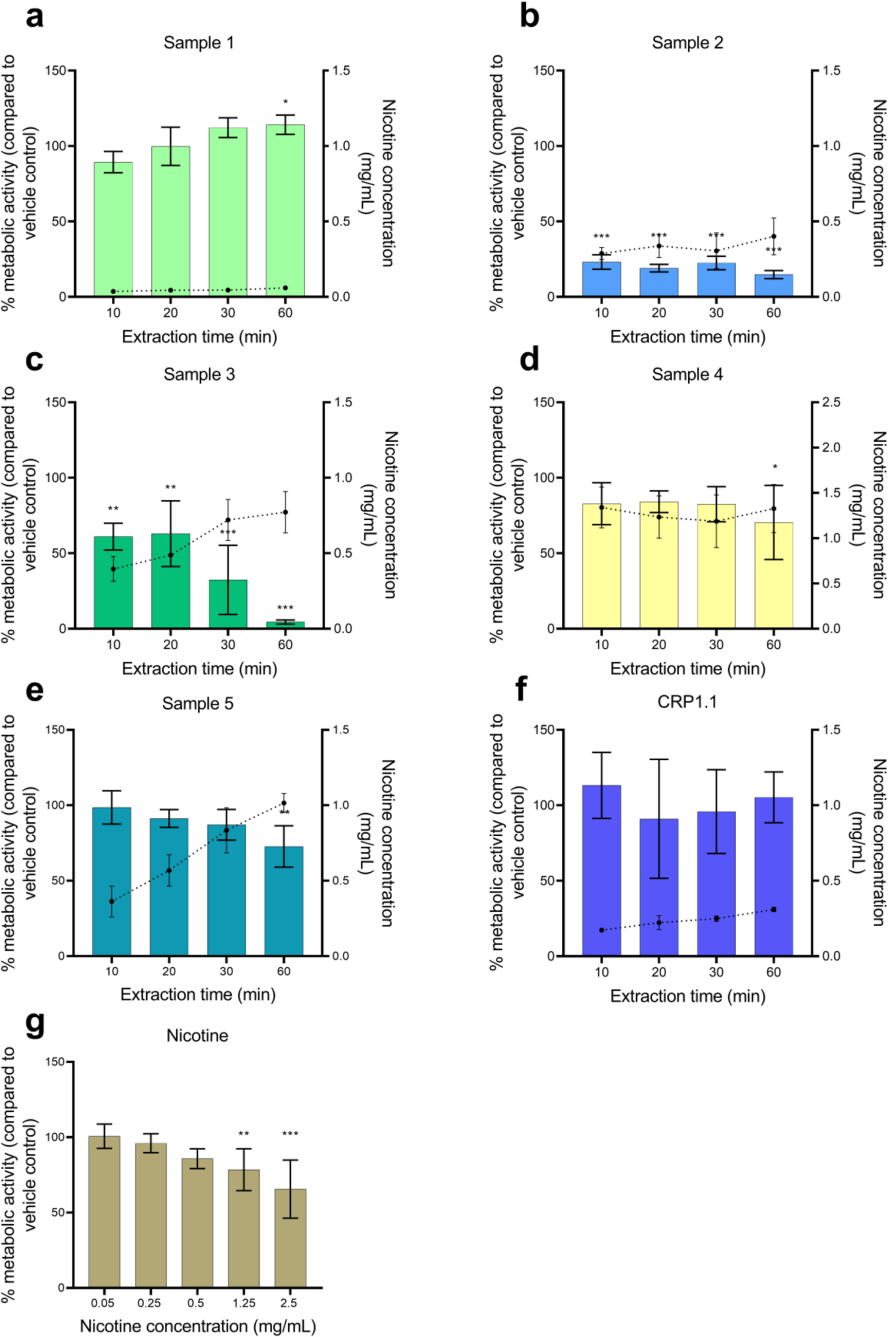
Metabolic activity measured after a 24-h exposure period of human gingival fibroblasts (HGF-1) to **a–e** nicotine pouch extracts, **f** reference snus CRP1.1 and **g** nicotine control in dissolution medium. Nicotine concentrations of sample extracts are represented through the dotted line, plotted against the secondary Y-axis. Results are presented as mean of triplicates and standard deviation. *p < 0.05, **p < 0.01, ***p < .0.001

The metabolic activity of cells in response to a 24-h exposure to the sample extracts is shown in Fig. 1. The mean nicotine concentrations of the extracts after dilution for toxicity assays are included in the figures. The percentage metabolic activity is shown in comparison to the vehicle control. A statistically significant increase was observed for the 60 min extract of sample 1 (Fig. 1a). Extracts of sample 2 (0.29–0.40 mg/mL nicotine) and 3 (0.40–0.78 mg/mL nicotine) at all extraction times caused a statistically significant decrease in the metabolic activity of cells (Fig. 1b, c). The decrease of metabolic activity was dependent on extraction time for sample 3. For samples 4 and 5, only the extracts at 60 min (1.33 mg/mL and 1.02 mg/mL nicotine, respectively) caused a statistically significant decrease in metabolic activity (Fig. 1d, e). The metabolic activity of cells treated with nicotine at different concentrations significantly decreased at a concentration of 1.25 and 2.5 mg/mL (Fig. 1g). Reference snus CRP1.1 did not have an effect on the metabolic activity of HGF-1 cells (Fig. 1f).

**Fig. 2:**
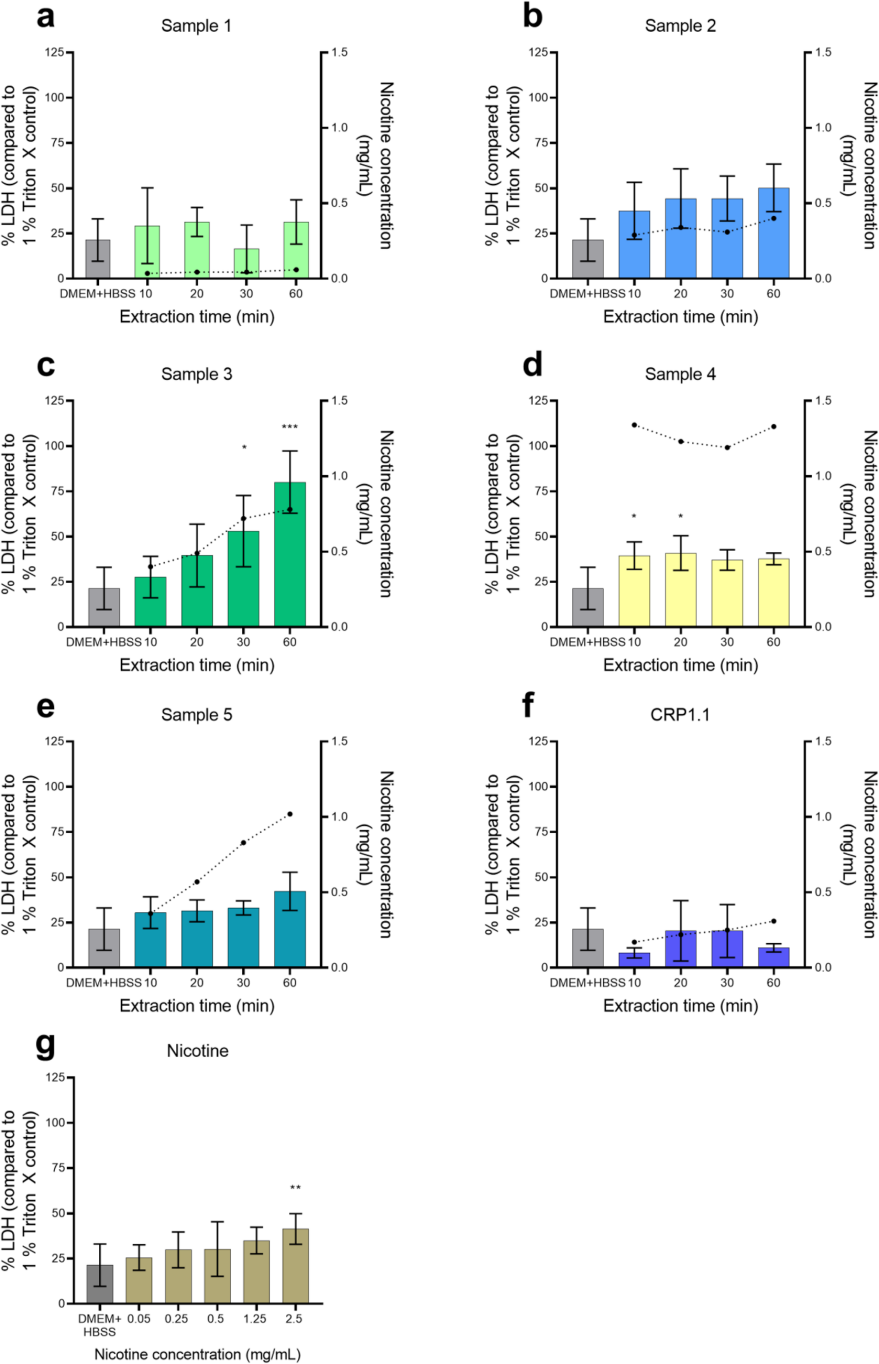
Release of lactate dehydrogenase (LDH) measured after a 24-h exposure period of human gingival fibroblasts (HGF-1) to **a–e** nicotine pouch extracts, **f** reference snus CRP1.1, and **g** nicotine control in dissolution medium. Nicotine concentrations of sample extracts are represented through the dotted line, plotted against the secondary Y-axis. Results are presented as mean of triplicate and standard deviation. *p < 0.05, **p < 0.01, ***p < 0.001

Figure 2 shows the results for the LDH release from the cells after a 24-h exposure to the sample extracts compared to the vehicle control. The increase in LDH levels was statistically significant for the 30 and 60 min extracts of sample 3 (Fig. 2c), the 10 and 20 min extracts of sample 4 (Fig. 2d), and at the highest concentration of the nicotine control with 2.5 mg/mL (Fig. 2g). In general, the results are consistent with the observations for metabolic activity, although not as pronounced.

### Oxidative stress

**Fig. 3:**
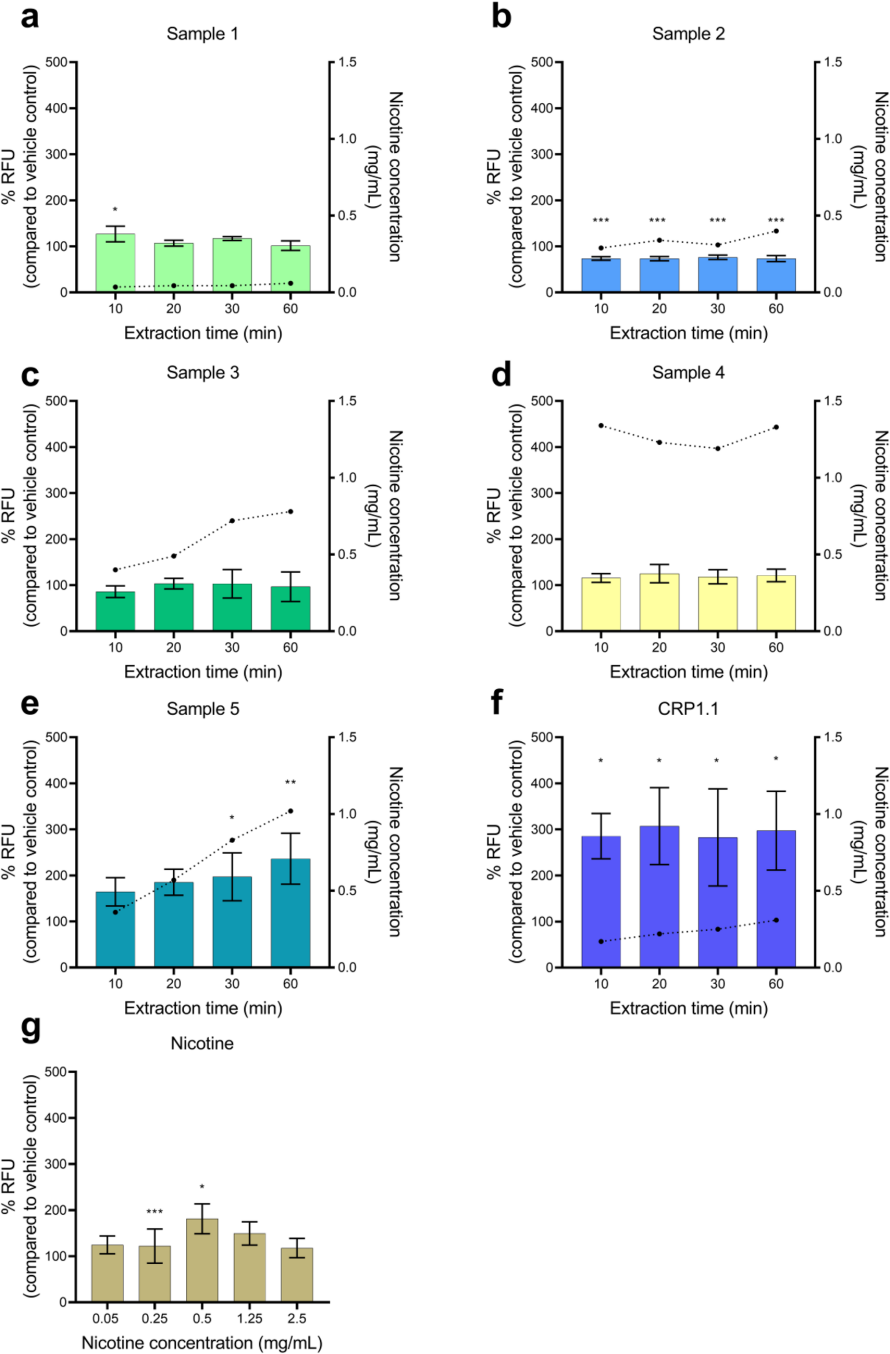
Reactive oxygen species (ROS) measured using the 2’,7’-dichlorofluorescin diacetate (DCFDA) assay after a 4-h exposure period of human gingival fibroblasts (HGF-1) to **a–e** nicotine pouch extracts, **f** reference snus CRP1.1, and **g** nicotine control in dissolution medium. Nicotine concentrations of sample extracts are represented through the dotted line, plotted against the secondary Y-axis. Results are presented as mean of triplicate and standard deviation. *p < 0.05, **p < 0.01, ***p < 0.001

The results of the DCFDA assay as a measure of cellular oxidative stress are shown in Fig. 3. When compared to vehicle control, increased ROS formation was observed for the 30 and 60 min extracts of sample 5 (Fig. 3e), the 10 min extract of sample 1 (Fig. 3a), all CRP1.1 extracts (Fig. 3f), and for doses of 0.5 mg/mL and 1.25 mg/mL of nicotine (Fig. 3g). Conversely, sample 2 significantly reduced ROS formation at all measured time points (Fig. 3b), whereas samples 3 and 4 produced no measurable alterations of ROS levels (Fig. 3c, d) compared with the vehicle control.

### Expression of genes related to oxidative stress and inflammation

**Fig. 4:**
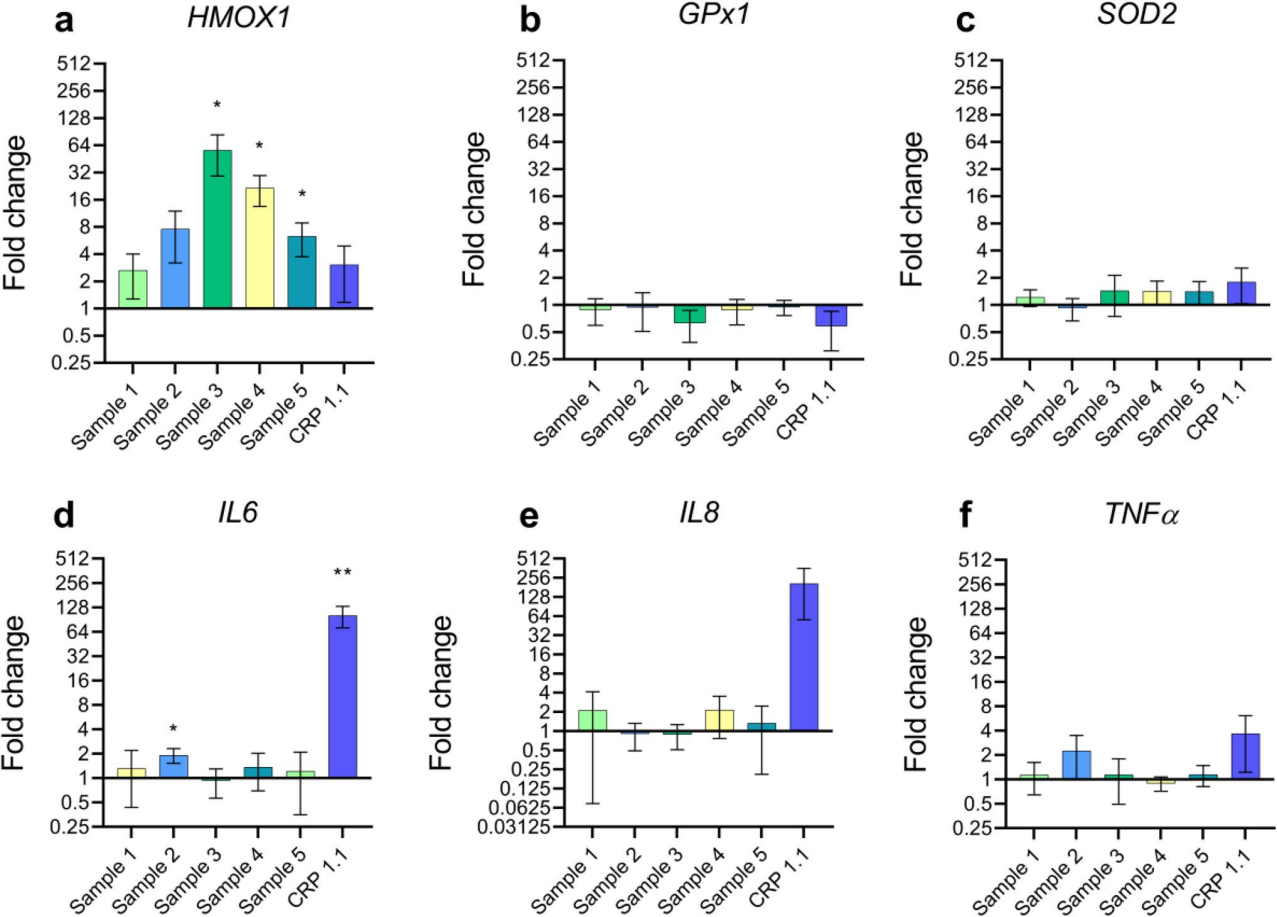
Quantitative real-time polymerase chain reaction (qRT-PCR) data in response to a 4-h exposure period to the 20 min sample extracts. Fold changes are calculated in comparison to the vehicle control. β-Actin (ACTB) served as the housekeeping gene. Results are presented as mean of triplicate and standard deviation in a log (2) scale. *p < 0.05, **p < 0.01, ***p < 0.001

For the measurement of gene expression, only the 20 min extracts were used. Gene expression of the antioxidant gene *HMOX1* was significantly upregulated after exposure to the extracts of samples 3, 4, and 5 (Fig. 4a). Expression of the pro-/anti-inflammatory gene *IL6* was upregulated by sample 2 and reference snus CRP1.1 (Fig. 4d). Effects on the regulation for the antioxidant genes *GPx1* (Fig. 4b) and *SOD2* (Fig. 4c) and the pro-inflammatory genes *IL8* and *TNFα* were not statistically significant. For CRP1.1, a trend towards IL8 increase was visible (Fig. 4e) which did not reach statistical significance with the statistical analysis applied.

### Osmolarity of sample extracts

Osmolarity was found in the range of about 250 mOsm/kg for all nicotine pouch extracts generated (see Supplementary Information Fig. 2). No differences were observed either between pouches or between different extraction time points of one pouch. Reference snus CRP 1.1 showed a higher osmolarity of 300 – 350 mOsm/kg. Osmolarity of the medium control and vehicle control was approximately 230 mOsm/kg.

### Morphological changes in HGF-1

Distinct morphological alterations of HGF-1 cells were observed 24 h after exposure to 1.25 and 2.5 mg/mL nicotine, as well as with samples 2, 3, 4 and 5 (see Supplementary Information Figs. 7, 8, 10—13).

### Flavor components detected in nicotine pouches

Table 1 depicts the five pouches with their classification into flavor categories according to the proposal of Krüsemann et al. (2019) in the case of e-liquids. In addition, Table 1 also shows the flavor compounds detected. The screening of the nicotine pouches for flavor components resulted in the detection of 53 substances of which 17, 16, 24, 23, and 12 substances were identified in sample 1, 2, 3, 4, and 5, respectively. Of these 53 substances, seven (benzyl alcohol, benzaldehyde, benzyl benzoate, carvone, citral, limonene, and linalool) received a harmonized hazard classification label according to the Classification, Labelling and Packaging (CLP) Regulation (EC) No 1272/2008 (European Parliament and the Council of the European Union 2008). In each pouch, at least two substances were identified with such a harmonized classification. Benzyl alcohol, for instance, is classified as a compound belonging to acute oral and inhalation toxicity category 4. Benzaldehyde and benzyl benzoate are classified as acute oral toxins of category 4. Carvone, citral, and limonene are classified as skin sensitizers of category 1. In addition, citral and limonene are classified as skin irritants of category 2. Linalool is classified as skin sensitizer of category 1B.

**Table 1:** Flavor compounds identified in five nicotine pouches using GC/MS

Sample No	Flavor category (Krusemann et al. 2019)	Identified compounds (hazard statements according to CLP)
1.	Spices–Chili	α-terpineol, β-terpineol, benzyl alcohol (H302—Cat. 4^1^, H332—Cat. 4^4^), bornyl acetate, carvone (H317—Cat. 1)^3^, caryophyllene, cinnamaldehyde, citral (H315—Cat. 2^2^, H317—Cat. 1^3^), dihydrocapsaicin, eugenol, geranyl acetate, limonene (H315—Cat. 2^2^, H317—Cat. 1^3^), linalool (H317—Cat. 1B)^3^, menthol, n-hexadecanoic acid, tris(2-butoxyethyl) phosphate, terpinen-4-ol
2.	Other beverages–Cola	α-terpineol, β-terpineol, 1-terpinenol, 1,4-cineole, benzyl alcohol (H302—Cat. 4^1^, H332—Cat. 4^4^), benzyl benzoate (H302—Cat. 4^1^), camphene, carvone (H317—Cat. 1)^3^, eucalyptol, isopulegol, linalool (H317—Cat. 1B)^3^, menthol, neryl acetate, octanal propylene glycol acetale, tris(2-butoxyethyl) phosphate, terpinen-4-ol
3.	Tobacco	α-terpineol, β-bourbonene, cis-β-farnesene, β-terpineol, benzyl alcohol (H302—Cat. 4^1^, H332—Cat. 4^4^), benzaldehyde (H302—Cat. 4)^1^, carvone (H317—Cat. 1)^3^, carvyl acetate, cis-carveol, caryophyllene, dihydrocarvone, eucalyptol, humulene, isomenthyl acetate, isopulegol, limonene (H315—Cat. 2^2^, H317—Cat.1^3^), linalool (H317—Cat. 1)^3^, menthol, menthone, neomenthol, neomenthyl acetate, piperitone, pulegone, terpinen-4-ol
4.	Menthol	α-terpineol, β-pinene, γ-elemene, 3-methyl cyclohexanone, artemisia triene, β-bourbonene, butyl palmitate, butylated hydroxytoluene, carvone (H317—Cat. 1)^3^, caryophyllene, eucalyptol, dihydrocarvone, isomenthol, isomenthyl acetate, isopulegol, limonene (H315—Cat. 2^2^, H317—Cat. 1^3^), linalool (H317—Cat. 1)^3^, menthol, menthone, myosmine, piperitone, pulegone, tris(2-butoxyethyl) phosphate
5.	Other beverages–Energy	β-ionone, β-pinene, 1,3-di-tert-butylbenzene, benzaldehyde propylene glycol acetate, benzyl alcohol (H302—Cat. 4^1^, H332—Cat. 4^4^), carvone (H317—Cat. 1)^3^, ethyl maltol, isopulegol, menthol, methyl anthranilate, raspberry ketone, vanillin

Compounds are listed alphabetically along with its hazard statement codes according to the Classification, Labelling and Packaging (CLP) Regulation (EC) No 1272/2008, where applicable. The hazard statements and the applicable categories of toxicity for the specific flavors are explained in the legend. The flavor categories were taken from Krusemann et al. (2019) and identified according to the flavor descriptions listed on the respective packaging

^1^H302 – Harmful if swallowed [Acute oral category 4 – Acute toxicity estimate (ATE*) between 300 and 2000 mg/kg bodyweight]

^2^H315 – Causes skin irritation [Skin irritation category 2 – production of reversible damage to skin following a 4 h skin exposure with 2 of 3 tested animals having a mean score between 2.3 and 4]

^3^H317 – May cause an allergic skin reaction [Skin sensitization Category 1 – when category 1A cannot be excluded but human data or animal data are not sufficient for a sub-categorization into category 1A or 1B

Skin sensitization Category 1B – low to moderate frequency occurrence in humans and/or from animal testing potential sensitization can be presumed]

^4^H332 – Harmful if inhaled [Acute inhalational category 4 – ATE between 10 and 20 mg/l]

*The ATE is derived from the LD50-values for the oral route and the LC50-values for the inhalational route

The underline is used for bettter visibility of compounds that have a CLP statement

In addition, the following seven compounds did not have any authorization as food flavorings on the European market: tris-(2-butoxyethyl), cis-β-farnesene, humulene, isomenthyl acetate, pulegone, isomenthol, and myosmine.

The five pouches investigated strongly varied in their composition; just carvone and menthol were detected in all of these pouches.

## Discussion

Nicotine pouches do not contain tobacco leaf material. As such, they are newly emerging products on the market, but their local (buccal mucosa) and systemic health effects are still unknown. We performed this in vitro study to investigate the cytotoxic potential of five different nicotine pouches and the reference snus CRP1.1 in the human gingival fibroblast cell line HGF-1. The selected nicotine pouches covered a wide range of nicotine strengths (see Supplementary Information, Table 1) and different flavor compositions (Table 1). In this study, the cell biological endpoints cytotoxicity, altered expression of inflammatory and oxidative stress genes, as well as the total cellular oxidative stress levels were determined. In addition, nicotine concentrations in the sample extracts and the flavoring agents used in the pouches were identified to discuss their potential contribution to the toxic effects observed. Nicotine, at the concentrations present in the nicotine pouch extracts, did not appear to be the driving factor in any of the cellular effects observed.

Cytotoxicity in HGF-1 cells following a 24-h period of exposure was already inducible with extracts of pouches 2 and 3 that contained no more than 0.4 mg/mL and 0.7 mg/mL of nicotine, respectively. This level was far below the concentration required to induce cytotoxicity in the control experiment with pure nicotine (1.25 mg/mL), suggesting that compounds other than nicotine in the pouch extracts, such as flavorings, contributed, at least in part, to this cellular endpoint.

In their own studies, nicotine pouch manufacturers claimed that no cytotoxic effect was detectable when compared to the reference cigarette 1R6F, reference snus CRP1.1, or various competitor pouches (Bishop et al. 2020; East et al. 2021). In our study, CRP1.1 did not exhibit any cytotoxicity, although conflicting results had previously been published (Bishop et al. 2020; East et al. 2021; Zhao et al. 2021). Consistent with our findings, a recent study without industry involvement revealed nicotine pouches being more cytotoxic than snus in an oral epithelial cell line (HGEP) (Shaikh et al. 2022).

Inflammation and oxidative stress are two interrelated factors involved in the development of smoking- and tobacco-related diseases (Caliri et al. 2021). Here, we measured the expression of oxidative stress-related (*HMOX1, SOD2, *and* GPx1)* and inflammatory (*IL8, IL6, *and* TNFα)* marker genes. According to our results, nicotine pouch extracts induced an oxidative stress response rather than an inflammatory response in the treated cells. Conversely, CRP1.1 induced *IL6* and showed a trend towards *IL8* increase. In a study by a manufacturer, nicotine pouches did not induce upregulation of antioxidant genes (*Srxn1* and *Blvrb*), whereas CRP1.1 resulted in a 4 to 12 fold increase in expression levels (Bishop et al. 2020). Other researchers have previously shown that CRP1 upregulates *IL6* and *IL8* gene expression and is capable of inducing ROS (Zhao et al. 2021). Oxidative stress and inflammation might lead to adverse health effects at the site of nicotine pouch placement.

Other substances in nicotine pouches could contribute to cytotoxicity and altered gene expression as concentrations are likely to rise with an increasing extraction time likewise to nicotine. Aroma substances contained in the five pouches were screened in part 1 of this project (Mallock-Ohnesorg et al. 2023). Seven of the identified flavor compounds received a harmonized hazard classification label according to CLP: benzaldehyde, benzyl alcohol, benzyl benzoate, carvone, citral, limonene, and linalool. However, all of them are authorized by EFSA for its use as food additives and are generally recognized as safe by the FDA. Seven substances were not authorized as food flavorings in the European Union: tris-(2-butoxyethyl), pulegone, myosmine, cis-β-farnesene, humulene, isomenthyl acetate, and isomenthol.

Cytotoxic effects have been described in different cell lines beforehand for benzyl alcohol (Chang et al. 2008), benzaldehyde (Ulker et al. 2013), citral (Mesa-Arango et al. 2009; Souza et al. 2020), d-limonene (Hajizadeh et al. 2019), linalool (Prashar et al. 2004), cinnamaldehyde (Behar et al. 2018), dihydrocapsaicin (Halme et al. 2016), and eugenol (Escobar-Garcia et al. 2016). These findings may suggest that flavors contributed to the pouch extract-mediated cytotoxicity seen in our study.

Other biological effects, such as altered gene expression and ROS production, as discussed above, might have been induced–at least partly–by flavors as well. In the present study, benzaldehyde was detected only in sample 3, which showed a trend towards downregulation of *GPx1* and dose-dependent cytotoxicity. However, it cannot be concluded that benzaldehyde is the only cause for the observed cytotoxicity as it could also be an effect of the flavoring mixture. Increased radical production was measured in aerosols of citral containing e-liquids (Reilly et al. 2018) and intracellular ROS production following treatment with different concentrations of citral (Sinha et al. 2014) or linalool (An et al. 2021) were described. Oxidation products of d-limonene and linalool are known skin sensitizers (Kim et al. 2013; Skold et al. 2004). It thus seems possible that these flavorings exert their sensitizing effects also in the oral mucosa following repeated nicotine pouch consumption. ROS production, cytotoxicity and skin irritation could lead to local adverse effects in nicotine pouch users at the site of pouch placement. Local irritation indeed has been reported by nicotine pouch users (Shao et al. 2022).

Substances that do not belong to the group of authorized food flavorings in the EU are discussed more detailed in part 1 (Mallock-Ohnesorg et al. 2023). Briefly, pulegone, myosmine, isomenthyl acetate, and isomenthol could be impurities from extraction processes. Cis-β-farnesene, humulene, and tris(2-butoxyethyl)phosphate are no flavoring substances. The latter compound is used as a flame retardant and could stem from the pouch material.

Further, terpenes, such as menthol, d-limonene, linalool, and carvone, facilitate dermal absorption of other compounds often used in topical drug delivery (Aqil et al. 2007). It was also shown that epidermal absorption of nicotine in e-liquid refills occurs faster once limonene is being added (Frasch and Barbero 2017). Therefore, the addition of flavorings in nicotine pouches might not only contribute to product’s attractiveness, it also likely leads to an accelerated nicotine absorption through the buccal mucosa and thus to the induction of addictiveness.

In this study, we showed that aqueous extracts of nicotine pouches adversely affect human gingival cells in culture with respect to cytotoxicity, induction of intracellular ROS, and the expression of inflammatory genes. Among all effects detected, only the morphological alterations can be solely attributed to nicotine, an observation already described before (Kang et al. 2011; Takeuchi-Igarashi et al. 2016). However, the mechanisms and health implications of these nicotine-induced morphological changes are not yet understood (Kang et al. 2011; Takeuchi-Igarashi et al. 2016).

Taken together, our results demonstrate that nicotine pouches exert biological activities. Depending on the endpoints investigated, nicotine-independent and -dependent effects can be observed. In addition, and based on our data, a synergistic effect of nicotine and other ingredients such as flavorings in cells is also likely. In the context of e-cigarettes, flavorings were shown to have cytotoxic effects on various cell lines (Behar et al. 2018; Hua et al. 2019). In our study, 53 different substances, including 46 flavoring agents, were found in the nicotine pouches. The cytotoxic effects on mucosal cells found in this study suggest that local lesions are likely to occur after repeated product use. While our study on nicotine pouch flavorings should provide a starting point, further investigation is needed to identify the specific flavorings responsible for the observed effects. First, of the flavorings identified in the first part of the study (Mallock-Ohnesorg et al. 2023), potentially hazardous compounds, particularly the suspects discussed here, should be quantified in nicotine pouches. Second, quantities present in pouches should be compared with dose–response curves for toxicological endpoints in relevant cell lines. However, the toxicity of the mixtures needs to be considered in further studies as well, since an interplay of nicotine and multiple flavorings is possible. The results presented here provide initial insights to tailor future studies to compounds and endpoints relevant for the assessment of nicotine pouches.

## Supplementary Information

Below is the link to the electronic supplementary material.

Supplementary file1 (DOCX 3146 KB)

## Data Availability

The datasets generated during and/or analysed during the current study are available from the corresponding author on reasonable request.
