# Supplementary material for: Oral nicotine pouches with an aftertaste? Part 2: in vitro toxicity in human gingival fibroblasts
Source: Arch Toxicol. 2023 Jul 23;97(9):2343–56. doi: 10.1007/s00204-023-03554-9 (PMC10404181; doi:10.1007/s00204-023-03554-9)
Supplement: Supplementary file 1 — Supplementary file1 (DOCX 3146 KB) [file 204_2023_3554_MOESM1_ESM.docx]

**Supplementary Material**

**Oral nicotine pouches with an aftertaste? Part 2: In vitro toxicity in human gingival fibroblasts**

Selina Rinaldi^*1,2^, Elke Pieper^1^, Thomas Schulz^1^, Ralf Zimmermann^2^, Andreas Luch^1^, Peter Laux^1^, Nadja Mallock-Ohnesorg^1^

^1^ German Federal Institute for Risk Assessment (BfR), Department of Chemical and Product Safety, 10589 Berlin, Germany

^2^ Chair of Analytical Chemistry, Joint Mass Spectrometry Centre, University of Rostock, 18059 Rostock, Germany

*Corresponding author: Selina.Rinaldi@bfr.bund.de

**Table of contents**

**1.** **METHODS** 2

**1.1** **Nicotine pouch samples** 2

**1.2** **Method validation** 2

**1.3** **Example chromatogram** 3

**1.4** **Primer sequences** 4

**2** **RESULTS** 5

**2.1** **Osmolarity** 5

**2.2** **Morphology of human gingival fibroblasts (HGF-1)** 6

### **METHODS**

## **Nicotine pouch samples**

Supplementary Table 1 shows the 15 nicotine pouches that were used in the study, including information on nicotine content and flavor group. The nicotine contents were analyzed in a previous study using gas chromatography with flame ionization (Mallock et al. 2022). Due to the legal assignments, the German Federal Institute of Risk Assessment (BfR) is not allowed to reveal company names which would lead to a comparison of specific products which was also not the aim of this study.

**Supplementary Table 1** Information on nicotine pouches used for in vitro nicotine dissolution

| **Sample number** | **Nicotine content (mg/pouch)** |
| --- | --- |
| 1 | 3.8 |
| 2 | 11.7 |
| 3 | 17.2 |
| 4 | 27.0 |
| 5 | 47.4 |
| 6 | 20.2 |
| 7 | 4.6 |
| 8 | 1.8 |
| 9 | 43.7 |
| 10 | 29.4 |
| 11 | 10.4 |
| 12 | 11.4 |
| 13 | 20.5 |
| 14 | 5.2 |
| 15 | 4.8 |

## **Method validation**

Nicotine concentrations of nicotine pouch extracts were determined using liquid chromatography with UV detection (LC-DAD). The quantification method was validated for the following parameters: linearity, accuracy, precision, storage stability, and limits of detection (LOD) and quantification (LOQ) (Supplementary Table 2). Quality control samples were generated in matrix using nicotine pouches with low nicotine content (approx. 2 mg nicotine/pouch). The pouches were extracted in 100 mL of extraction medium as described in the main text. Two matrix fraction, 1 and 2, were sampled after 10 and 60 min, respectively. One nicotine pouch was used for each matrix section. 5 and 10 µg/mL nicotine were added to matrix fraction 1. 100 and 300 µg/mL nicotine were added to matrix fraction 2 to obtain quality control samples. For each concentration, six quality control samples were produced and analyzed. Nicotine concentration of the matrix fractions without spiked nicotine were analyzed in triplicates. The mean nicotine concentrations of unspiked matrix fractions were subtracted from the nicotine concentrations in quality control samples to determine accuracy and precision.

**Supplementary Table 2** Summary of method validation for determination of nicotine from nicotine pouch extracts using LC-DAD

| **Parameter** | **Determined via** | **Acceptance criteria** | **Summary of results** |
| --- | --- | --- | --- |
| Working range and linearity | Regression coefficient | 0.5–1000 µg/mL  R² > 0.995 | 0.5 – 1000 µg/mL  R² > 0.9992 |
| Accuracy | Recovery of 4 different quality control samples in sextuplets on 2 days | Recovery:  80 – 120% | Mean recoveries, day 1:  5 µg/mL: 86.5%  10 µg/mL: 84.4%  100 µg/mL: 100.1%  500 µg/mL: 98.5%  Mean recoveries, day 2:  5 µg/mL: 83.8%  10 µg/mL: 84.0%  100 µg/mL: 99.1%  500 µg/mL: 97.4% |
| Precision | Standard deviation of six quality control measurements on 2 days | Standard deviation < 15% | Mean standard deviation, day 1:  5 µg/mL: 5.3%  10 µg/mL: 2.9%  100 µg/mL: 0.9%  500 µg/mL: 1.6%  Mean standard deviation, day 2:  5 µg/mL: 6.5%  10 µg/mL: 3.0%  100 µg/mL: 0.4%  500 µg/mL: 1.2% |
| Storage stability | Recovery of quality control samples after storage at +4 °C for 1 week. | Recovery:  80 – 120% | Mean recovery, after 1 week:  5 µg/mL: 85.5%  10 µg/mL: 85.5%  100 µg/mL: 100.3%  500 µg/mL: 98.2% |
| Limits of detection and quantification | Calibration method according to DIN 32645:2008(DIN ISO 32645:2008-11 2008) |  | Limit of detection:  0.042 µg/mL  Limit of quantification:   - 1. µg/mL |

## **Example chromatogram**

Supplementary Figure 1 shows an example chromatogram for nicotine measured in nicotine pouch extracts produced according to the developed dissolution protocol (see main text). The sample chromatogram shows the nicotine peak from sample extract 4 at 5 min with a measured concentration of 52.3 µg/mL.

**Supplementary Figure 1** Example chromatogram for nicotine detection in sample extract 4 at 5 min with a measured concentration of 52.3 µg/mL

## **Primer sequences**

The primer sequences shown in Supplementary Table 3 were used for the gene expression measurements using qRT-PCR. Beta-Actin (*ACTB*) served as the housekeeping gene.

**Supplementary Table 3** Primer sequences used for qPCR experiments

| **Target** | **Forward primer (‘5 -> ’3)** | **Reverse primer (‘5 -> ’3)** |
| --- | --- | --- |
| *ACTB* | CACCATTGGCAATGAGCGGTTC | AGGTCTTTGCGGATGTCCACGT |
| *IL8* | TCAGAGACAGCAGAGCACAC | GGCAAAACTGCACCTTCACA |
| *IL6* | ATAACCACCCCTGACCCAAC | CCCATGCTACATTTGCCGAA |
| *HO1* | AGAAGAGGCCAAGACTGCGTT | GGTCCTTGGTGTCATGGGTCA |
| *GPx1* | CCGACCCCAAGCTCATCA | GAAGCGGCGGCTGTACCT |
| *SOD2* | TTGGCCAAGGGAGATGTTAC | AGTCACGTTTGATGGCTTCC |
| *TNFa* | CTTCTGCCTGCTGCACTTTGGAG | GGCTACAGGCTTGTCACTCGG |

# **RESULTS**

## **Osmolarity**

**Supplementary Figure 2** Osmolarity of nicotine pouch extracts, CRP1.1, medium control and vehicle control. Nicotine pouch extracts and CRP1.1 were diluted with cell culture medium 1:1

Supplementary Figure 2 shows osmolarity of each sample extract (10, 20, 30, 60 min) diluted with cell culture medium 1:1. Osmolarity was approximately 250 mOsm/kg with no difference observed between pouches or time points. Slightly higher osmolarity was observed for CRP 1.1 reference snus. No difference between medium control and vehicle control was observed.





## **Morphology of human gingival fibroblasts (HGF-1)**

After a 24-h exposure to vehicle control and nicotine up to 1.25 mg/mL cells had their normal morphology (Supplementary Figure 3 – Supplementary Figure 6). Exposure to 1.25 mg/mL and 2.5 mg/mL nicotine (Supplementary Figure 7 + Supplementary Figure 8) as well as to all sample extracts (Supplementary Figure 10 - Supplementary Figure 13) but sample 1 (Supplementary Figure 9) and CRP1.1 (Supplementary Figure 14) led to a changed morphology with vacuoles inside the cells. Sample 1 and CRP1.1 had lower nicotine contents as the other samples. Vacuole formation appeared independent on cytotoxicity as the 10-minute extract of pouch 4 and 30-minute extract of pouch 5 did not have cytotoxic effects on HGF-1 cells.


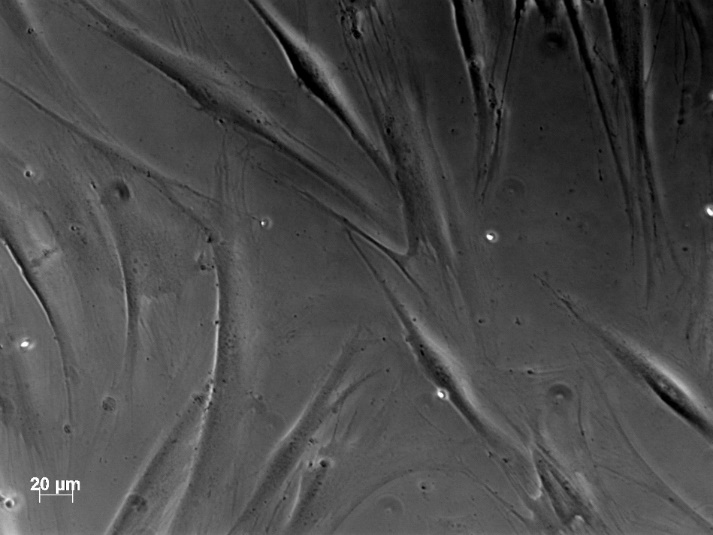


**Supplementary Figure 3** Microscopic picture of human gingival fibroblasts (HGF-1) after a 24-h exposure to vehicle control (magnification 20X)


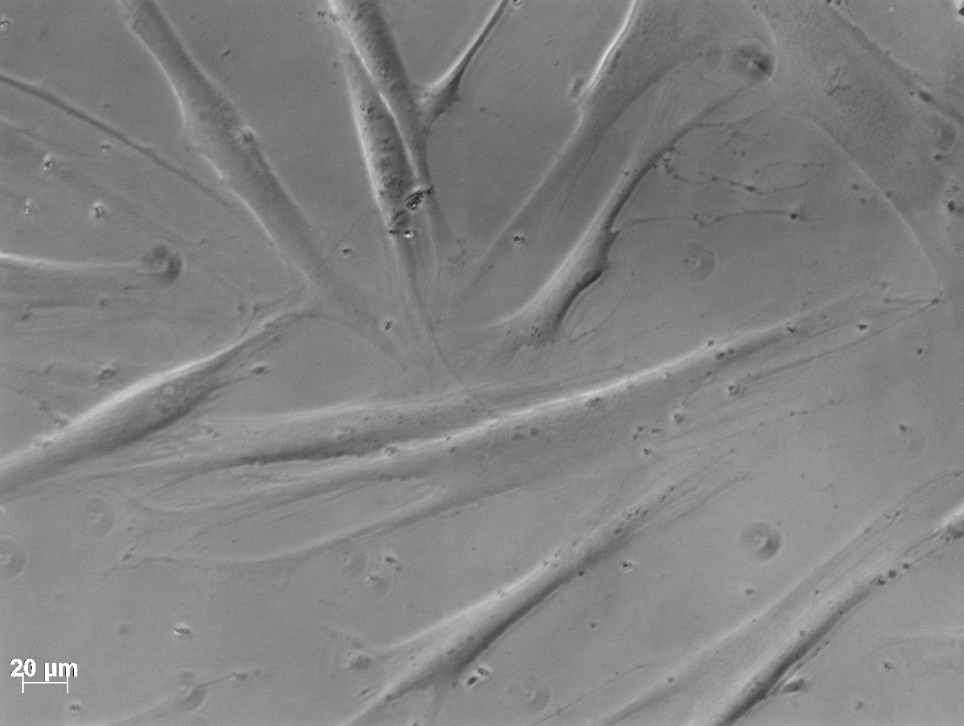


**Supplementary Figure 4** Microscopic picture of human gingival fibroblasts (HGF-1) after a 24-h exposure to 0.05 mg/mL nicotine control (magnification 20X)


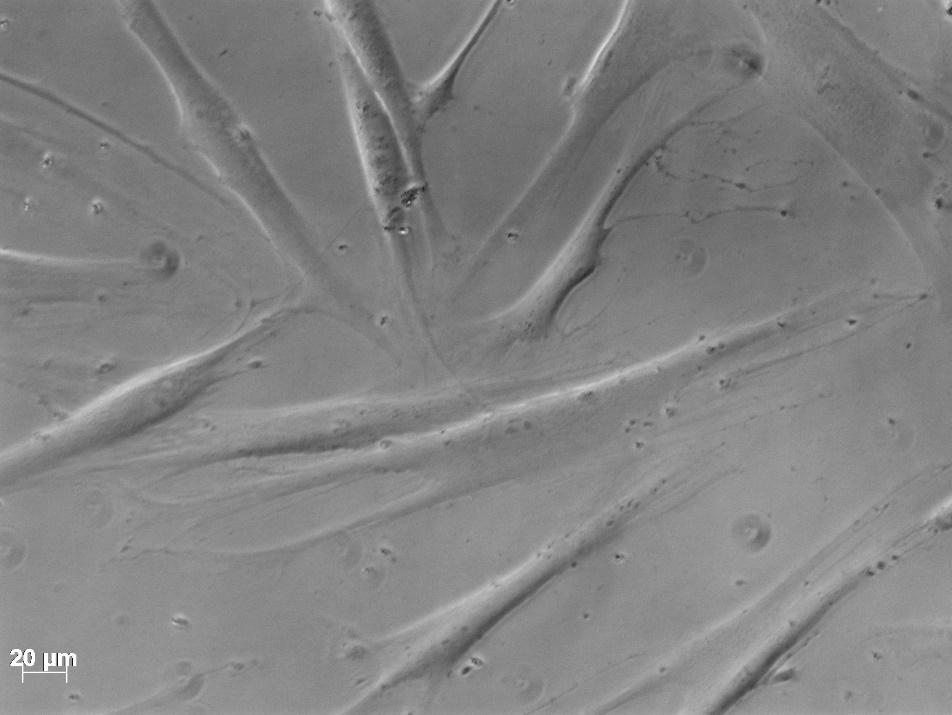


**Supplementary Figure 5** Microscopic picture of human gingival fibroblasts (HGF-1) after a 24-h exposure to 0.25 mg/mL nicotine control (magnification 20X)


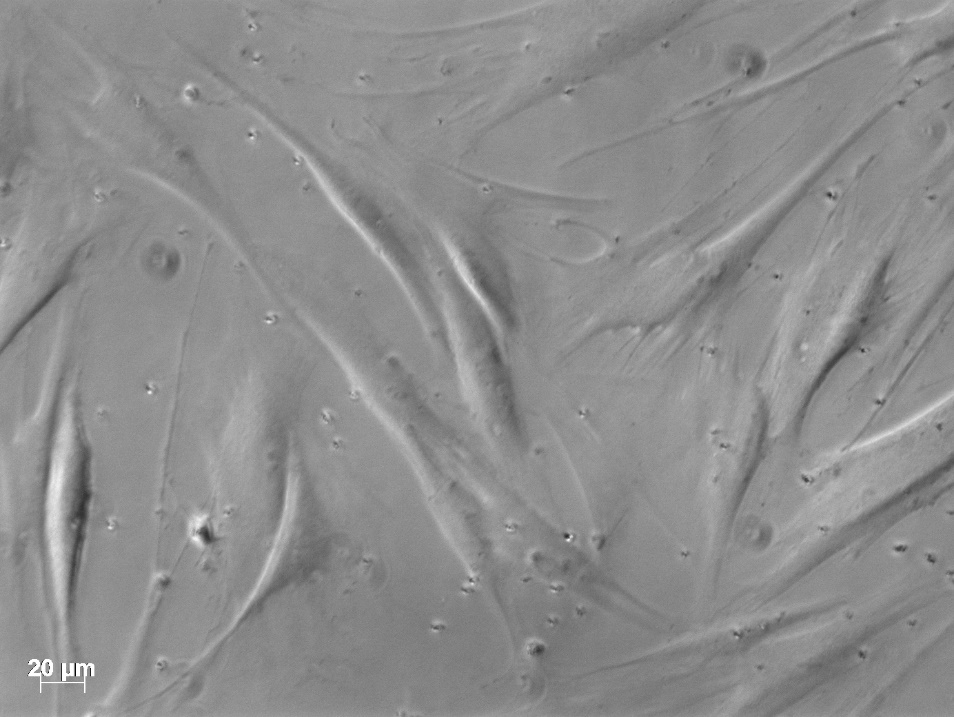


**Supplementary Figure 6** Microscopic picture of human gingival fibroblasts (HGF-1) after a 24-h exposure to 0.5 mg/mL nicotine control (magnification 20X)


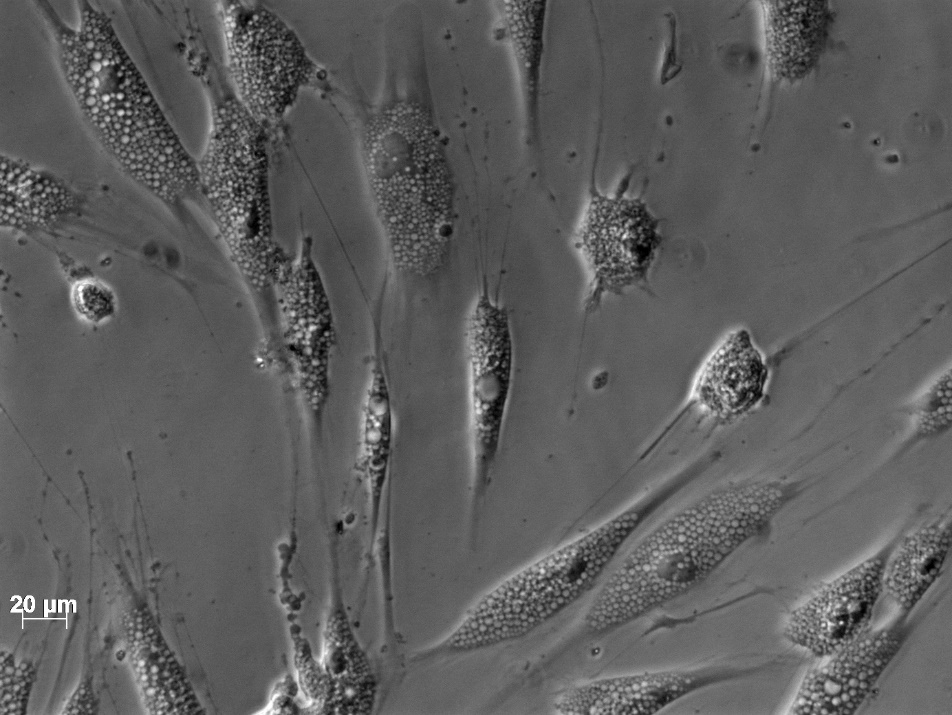


**Supplementary Figure 7** Microscopic picture of human gingival fibroblasts (HGF-1) after a 24-h exposure to 1.25 mg/mL nicotine control (magnification 20X)


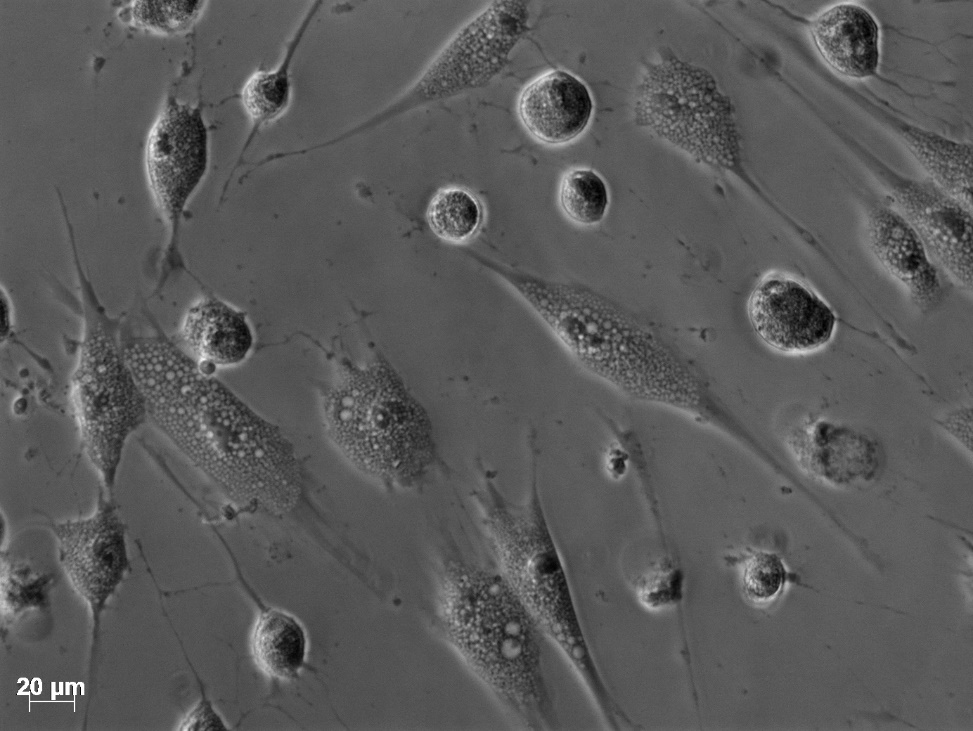


**Supplementary Figure 8** Microscopic picture of human gingival fibroblasts (HGF-1) after a 24-h exposure to 2.5 mg/mL nicotine control (magnification 20X)

**
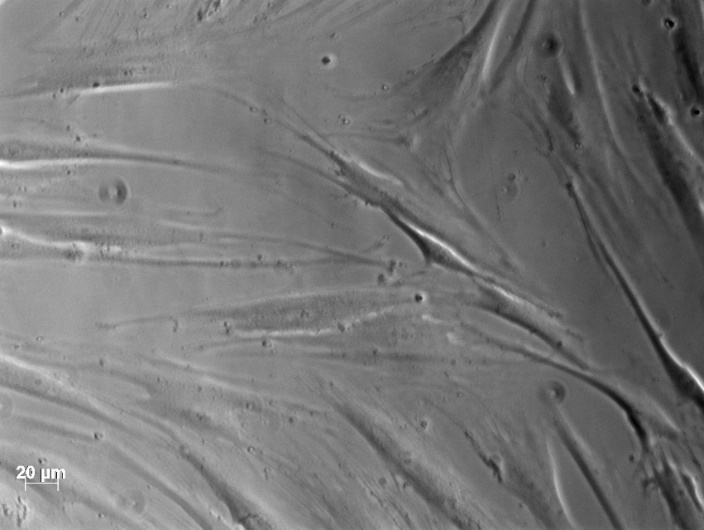
**

**Supplementary Figure 9** Microscopic picture of human gingival fibroblasts (HGF-1) after a 24-h exposure to the 10 min pouch extract 1 (magnification 20X)


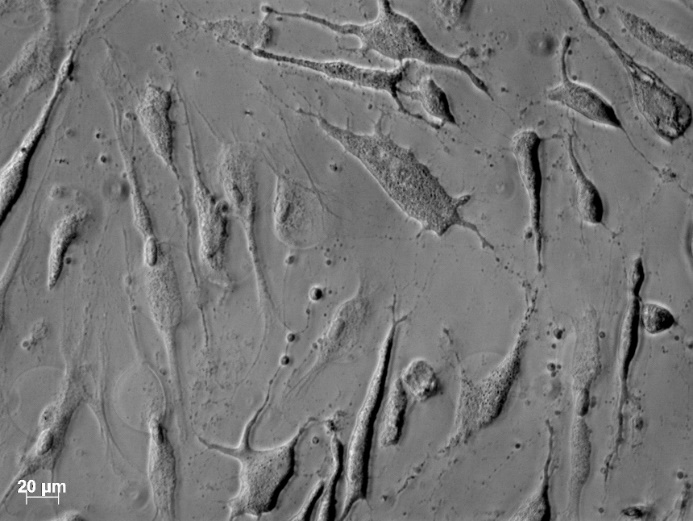


**Supplementary Figure 10** Microscopic picture of human gingival fibroblasts (HGF-1) after a 24-h exposure to the 30 min pouch extract 2 (magnification 20X)


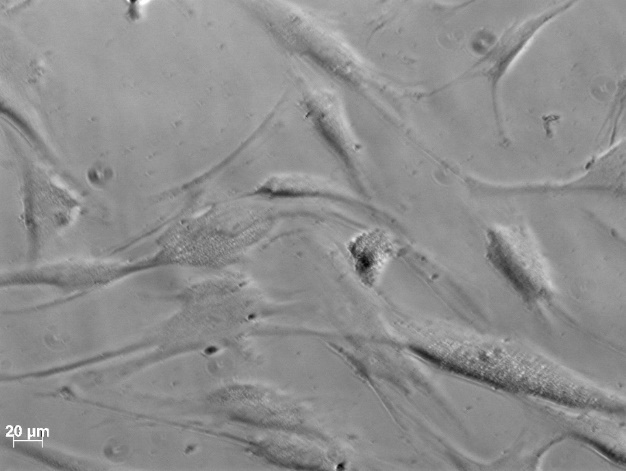


**Supplementary Figure 11** Microscopic picture of human gingival fibroblasts (HGF-1) after a 24-h exposure to the 30 min pouch extract 3 (magnification 20X)


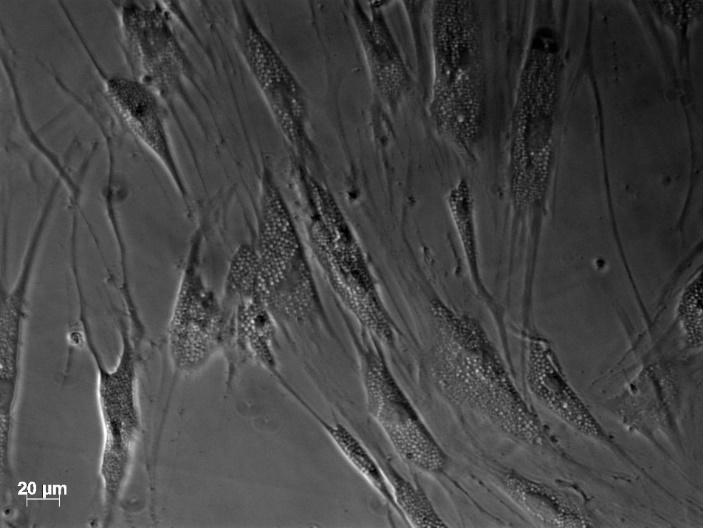


**Supplementary Figure 12** Microscopic picture of human gingival fibroblasts (HGF-1) after a 24-h exposure to the 10 min pouch extract 4 (magnification 20X)


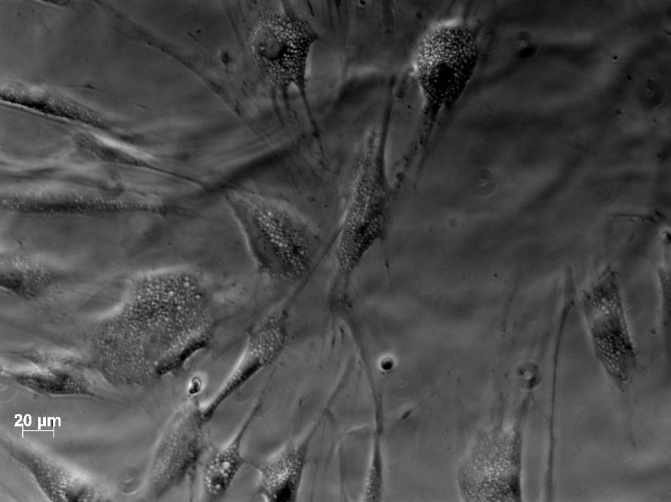


**Supplementary Figure 13** Microscopic picture of human gingival fibroblasts (HGF-1) after a 24-h exposure to the 30 min pouch extract 5 (magnification 20X)


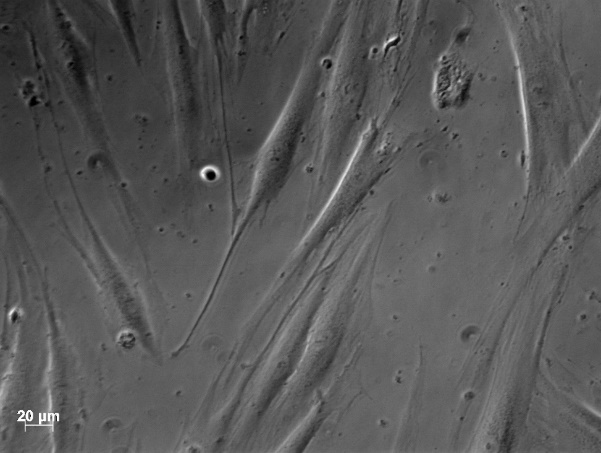


**Supplementary Figure 14** Microscopic picture of human gingival fibroblasts (HGF-1) after a 24-h exposure to the 60 min CRP1.1 extract (magnification 20X)

**References**

DIN ISO 32645:2008-11 (2008) Chemical analysis - Decision limit, detection limit and determination limit under repeatability conditions - Terms, methods, evaluation.

Mallock N, Schulz T, Malke S, Dreiack N, Laux P, Luch A (2022) Levels of nicotine and tobacco-specific nitrosamines in oral nicotine pouches. Tob Control doi:10.1136/tc-2022-057280
